# The prevalence of hearing impairment within the Cape Town Metropolitan area

**DOI:** 10.4102/sajcd.v63i1.105

**Published:** 2016-04-08

**Authors:** Lebogang Ramma, Ben Sebothoma

**Affiliations:** 1 Division of Communication Sciences & Disorders, University of Cape Town, South Africa; 2 Clinical Educator, Division of Communication Sciences & Disorders, University of Cape Town, South Africa

## Abstract

**Background:**

There is a lack of data on the prevalence of hearing impairment in South Africa. Current data is unreliable as it is based on national census information which tends to underestimate the prevalence of hearing impairment.

**Aim:**

The aim of this study was to estimate the prevalence of hearing impairment in the Cape Town Metropolitan area and to determine factors associated with hearing impairment.

**Method:**

A cross-sectional household survey involving 2494 partcipants from 718 households was conducted between the months of February and October 2013. Random cluster sampling was used to select four health sub-districts from eight health sub-districts in the Cape Town Metropolitan area using a method of probability proportional to size (PPS). The survey was conducted according to the World Health Organization (WHO) Ear and Hearing Disorders Survey Protocol and the classifcation of hearing impairment matched the WHO’s criteria for the grading of hearing impairment.

**Results:**

The overall prevalence of hearing impairment in the population of this study was 12.35% (95% CI: 11.06% – 13.64%) and prevalence of disabling hearing impairment was 4.57% (95% CI: 3.75% – 5.39%) amongst individuals ≥ 4 years old. The following factors were found to be associated with hearing impairment; male gender, age, hypertension, a history of head and neck trauma and a family history of hearing impairment.

**Conclusion:**

Based on the data from communities surveyed during this study, hearing impairment is more prevalent than previously estimated based on national population census information. Interventions for the prevention of hearing impairment in these communities should focus on individuals with associated risk factors.

## Introduction

Hearing is one of the essential senses for human communication. Hearing impairment at any stage of life can compromise the communication process and influence an individual’s quality of life (Gondim *et al.*, 2012). Hearing impairment in childhood can cause delays in the development of speech, language, and cognition which may later lead to educational disadvantage, social isolation and ultimately economic disadvantage (Finitzo, Albright & O’Neal, 1998; Stevens *et al.*, 2011; Yoshinaga-Itano, 2003). In adults, untreated hearing impairment has been linked to depression, anxiety and other psychological disorders (Kochkin & Rogin, 2000), as well as an increased risk of dementia (Lin *et al.*, 2011a).

Hearing impairment is a highly prevalent societal problem and it is one of the biggest contributors to the burden of disabilities in the world (Agrawal, Platz & Niparko, 2008). Hearing impairment was ranked first in the category of health conditions associated with disability and among the leading causes of the global burden of disability in the World Disability Report of 2011 (World Health Organization [WHO], 2011). The WHO estimates that approximately 360 million persons live with disabling hearing impairment worldwide and that the majority of these people live in developing countries, specifically South Asia, Asia Pacific and Sub-Saharan Africa (WHO, 2012).

Various risk factors, also linked to specific stages of life, are associated with hearing impairment. In children, risk factors for hearing impairment include, but are not restricted to: low birth weight, craniofacial anomalies, neonatal infections such as cytomegalovirus, herpes hyperbilirubinemia requiring exchange transfusion, respiratory distress, prolonged mechanical ventilation, meningitis, a family history of hearing loss and low Apgar score (Saunders *et al.*, 2007; Todd, 1994). For adults, the main risk factors for hearing impairment are advanced age and prolonged exposure to excessive noise (Isaacson & Vora, 2003). Research also links some non-communicable diseases, e.g. cardiovascular disease and/or diabetes, and lifestyle factors such as smoking with increased risk of developing hearing impairment during adulthood (Fransen *et al.*, 2008). Finally, exposure to ototoxic medications during the treatment of some health conditions such as cancer and drug-resistant tuberculosis have been shown to increase the risk of acquiring hearing impairment amongst both children and adults (Harris *et al.*, 2011; Saunders *et al.*, 2007; Whitehorn *et al.*, 2014).

Population-based surveys that estimate the magnitude of hearing impairment are generally few, despite the fact that hearing impairment is considered to be a leading contributor to the burden of disability (Stevens *et al.*, 2011). The lack of good estimates of the magnitude of hearing impairment makes it difficult to plan adequately for interventions and services aimed at individuals with hearing impairment. Currently, information on the prevalence of hearing impairment in South Africa is based primarily on data from the national population census (Statistics South Africa, 2003). Prevalence of hearing impairment estimates based on census data is typically based on self-report (or proxies’ report) of hearing impairment by individuals interviewed, and this tends to underestimate the magnitude of the problem (Nondahl *et al.*, 1998; Sindhusake *et al.*, 2001). This study therefore aims to estimate the prevalence of hearing impairment in the Cape Town Metropolitan area using a survey protocol that includes actual assessment of hearing status.

## Method

### Aim

The aim of this study was to estimate the prevalence of hearing impairment in the Cape Town Metropolitan area as well as to investigate the factors associated with hearing impairment in the population of this study.

### Research design

The study design made use of a cross-sectional population survey using the WHO’s Ear and Hearing Disorders Survey Protocol (WHO, 1999). The survey was conducted between the months of February and October 2013 in the Cape Town Metropolitan area.

### Context

The Cape Town Metropolitan area is one of the six health districts in the Western Cape. This health district is further divided into eight (health) sub-districts. The most recent national population census data of 2011 indicated that this metropolitan area has a population of 3 740 025 people evenly spread across these eight health sub-districts (Statistics South Africa, 2012).

### Participants

Participants in this survey included all individuals in the selected households who gave consent or assent to take part in this study. Minors (children younger than 12 years old) were only included in the study if their parents or legal guardians gave consent. For older children (≥ 12 years old) parental consent and assent from the child were obtained prior to being included in this study. Individuals were excluded from the study if they refused to give consent, were not at home after a second visit, were unable to understand either written or verbal instructions, or had cognitive impairment or communication difficulties.

### Sampling strategy

Random cluster sampling was used to select four sub-districts (cluster) from the eight health sub-districts within the Cape Town Metropolitan area through a method of probability proportional to size (PPS). An aerial map of each of the health sub-districts to be surveyed was used to aid in selecting the households to be sampled. The distribution of types of residences for each sub-district was obtained from the national census database to determine the proportion of each residence type within the health sub-district (Statistics South Africa, 2012). The sample was further stratified according to the type of residence (i.e. free standing house, block of flats and shacks). In residences which contained several households (e.g. main house and a backyard shelter), only one of the households per residence was surveyed. In the case of a block of flats, one floor per block of flats was surveyed and each unit/flat was treated as a household. For each residence type strata, a total of eight streets were randomly selected. Fifteen residences were identified in each street using the map, starting from the first residence to the right of the street corner and then including every second residence from this point until 15 households were visited for each of the eight selected streets.

### Sample size

The sample size for this study was determined using the WHO Ear and Hearing Disorders Survey Protocol (WHO, 1999). According to this protocol, assuming a prevalence of hearing impairment in the population of about 10%, with a precision of 1.08% and a design effect factor of 2, the required sample size for a simple random design (CI:95%) should be 5924 persons (WHO, 1999). At least 1693 households were required to be visited to obtain the required sample size using an average household size of 3.50 persons in the Cape Town Metropolitan area (Statistics South Africa, 2012).

## Research personnel

The research personnel comprised four teams of two senior Audiology students of the University of Cape Town per team, and two qualified audiologists with a total of 18 years of clinical experience between them. All of the students were senior Audiology students and therefore had adequate clinical skills required for the assessments required in this study. Furthermore, all the students were given additional training specific to the testing protocol used in this study to ensure consistency in assessment procedures and in the reporting of results.

### Ethical considerations

This study adhered to the ethical principles as outlined in the Declaration of Helsinki (World Medical Association Declaration of Helsinki, 2008). Informed consent was requested from potential participants prior to participation in this study and they were informed that their participation was voluntary. All efforts were made to safeguard the confidentiality of participants’ information and no specific identifying information was obtained for use in study reports. Participants were assured that they have the right to withdraw from this study at any time without negative consequences to themselves. Ethical approval to conduct the study was given by the Faculty Of Health Sciences Human Research Ethics Committee, University Of Cape Town (HREC REF 603/2012).

## Community entry

Key stakeholders in the communities selected for this study were approached by the researchers after ethical approval to conduct the study was granted. This served to inform the stakeholders about the study and to request their support in encouraging community members to participate in this study. Key stakeholders included: Managers of health facilities, representatives of community forums as well as representatives of community health forums in the selected sub-districts. Community members were additionally informed about the study through posters placed at various points in the community and through flyers placed in mail boxes.

### Pilot study

A pilot study was conducted in one of the four health sub-districts selected for this to test the study protocols. The outcome of the pilot study revealed that the assessment protocol proposed for this study was feasible in the communities being surveyed. Furthermore, the pilot study yielded information that optimised the efficient collection of data such as best test sequence when assessing participants’ hearing status and the best days of the weeks and times of the day to conduct the survey.

### Procedures

Research teams (in groups of two) approached selected households; explained the purpose of the study and procedures to be followed to the members of selected household with an invitation to participate. The research team entered the household and followed the following protocol, in households where the members (i.e. head of household or a guardian over the age of 18 years old) agreed and consented to be part of this study.

**Obtain consent:** The research team requested consent from each member of the household eligible to participate in this study. For young children (< 12 years old), the parent or legal guardian was asked to give consent on behalf of the child to participate in the study. For older children (≥ 12 years old, but ≤18 years old), assent and consent was obtained from the child before being tested. Participants were asked to sign a consent form (or assent form where applicable) to indicate their willingness to participate in the study.

**Assessment:** Audiometric assessments proceeded in the following sequence: measurement of ambient noise levels in the room, obtaining pertinent background history information (refer to the Ear form, Appendix 1), audiometric assessment, feedback and referral to the nearest health facility (where applicable). Participants ≥ 4 years were assessed using the following tests/procedures: otoscopic examination, tymapnometry and pure tone audiometry while partcipants aged 0–3 years were assessed using otoscopic examination, tymapnometry and Distortion Product Otoacoustic emissions (DPOAE) (refer to Appendix 2 for the detailed description of the assessment procedures).

## Classification of hearing loss

Hearing impairment was classified according to the WHO grading of hearing impairment (refer to Table 1-A2, Appendix 2) criteria. Disabling hearing impairment was defined as a permanent unaided hearing threshold level in the better ear of ≥ 41 dB HL (for adults) and permanent unaided threshold level in the better ear of ≥31 dB or (children younger than 15 years old) (WHO, 2014). Hearing impairment was further classified according to the type (conductive, mixed or sensorineural hearing loss) for all participants ≥ 4 years old.

## Reliability and validity

To ensure consistency and reliability of data collected, all members of the research team were trained on the study protocol and procedures prior to commencement of the study. Furthermore, the data collected was verified by re-visiting 10.7% of the selected households to confirm the following information: Head of the household (or the person who allowed the participants to enter the household), whether otoscopy, tympanometry and audiometry were done. The verification process showed good agreement (Kappa = 98%) between data collected by different research teams and therefore the data was considered was reliable. Finally, all decisions regarding the diagnosis of hearing loss were made by a qualified audiologist and all decisions involving a diagnosis of hearing loss were forwarded to the second qualified audiologist for cross-checking. There was also good agreement between the two audiologists (kappa = 0.96). All of the equipment used in this study were calibrated prior to the start of this study and also underwent a daily biological calibration check prior to data collection sessions. Test procedures used in this study were routine audiological tests with established validity.

Data was captured on the hard copy version of the WHO/PDH Ear and Hearing Disorders Examination Form (Version 7.1A), then transferred into an excel spread sheet by four research assistants. To ensure consistency when entering the data into an excel spread sheet, at least 10% of the data entered by each one of the research assistants was double checked by a different person to ensure accurate capturing of the data.

## Data analysis

Data analysis was performed using the STATA Data Analysis and Statistical Software package (Stata Corp LP, 2014) and both descriptive and inferential statistical methods were used. Tables and histograms were used to summarise and describe the results of the study. An independent *t*-test (*p* = 0.05) was used to compare hearing thresholds obtained under different ambient noise levels, whilst binomial logistic regression was used to model the relationship between hearing impairment and several explanatory variables; age, gender, level of education, a family history of hearing impairment, a history of stroke, a self-reported history of hypertension, a self-reported history of diabetes, smoking, a history of respiratory difficulties and a history of head and neck trauma. Binomial logistic regression was also done to estimate the probability (odds) of occurrence of hearing as a function of these explanatory variables.

## Results

### Demographic profile

A total of 791 households were approached and invited to take part in this study; 73 households either refused to participate (21 households) or no one was home on a second visit (52 households). It was not possible to find out the number of household members for 49 of the 73 households that were eligible but did not participate in this study. However, for the remaining 24 households in which household members were not assessed, the number of individuals per household ranged from 3 to 7. The number of non-respondent households was distributed evenly across all the health districts surveyed (i.e. there was no district with a markedly high number of non-respondent households).

**FIGURE 1:**
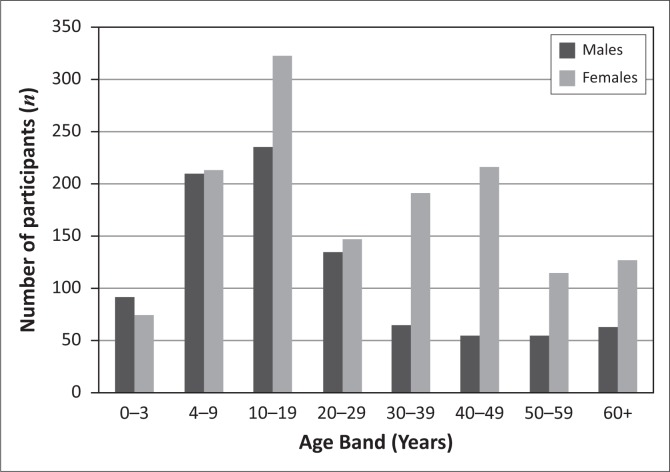
Age and gender profile of the participants (*n* = 2494).

Ultimately 718 households participated in this study and 2494 individuals (42% of the original study sample) from those households had their hearing screened; women comprised the majority of the participants (60.9%) and individuals in the 10–19 years old age category constituted 22.9% of the sample, whilst individuals 60+ years old constituted 7.6% of the sample (refer to Figure 1 for age and gender profile of participants in the study).

### Ambient noise levels

The average level of ambient noise during testing was 52.6±3.4 dBA. A *t*-test for independent samples (*p* = 0.05) showed no statistically significant relationship between the level of ambient noise during testing and the different categories of hearing impairment in this study (*p* = 0.163).

### Prevalence of hearing impairment

Of the total number of participants to this study, 12.35% (95% CI: 11.06% – 13.64%) had some degree of hearing impairment. See Table 1 for a detailed presentation of hearing impairment according to different age categories.

**TABLE 1:** Percentage (%) of participants by degree of hearing impairment and age category.

Disabling hearing impairment: Age band (Years)	*n*	No impairment	Slight impairment	Moderate	Severe	Profound
				
		0–25 dB (%)	26–30 or 40 dB (%)	31 or 41–60 dB (%)	61–80 dB (%)	≥ 81 dB (%)
0–3*	174	88.5	11.4	N/A	N/A	N/A
4–9	430	95.8	3.3	0.5	0.5	0.0
10–19	570	97.5	1.1	1.1	0.0	0.4
20–29	430	92.1	6.0	0.9	0.9	0.0
30–39	260	86.9	9.2	3.1	0.8	0.0
40–49	270	78.5	11.1	7.4	2.2	0.7
50–-59	170	67.1	22.4	7.1	2.4	1.2
60 +	190	61.1	21.1	12.6	4.2	1.1

**Total/Overall**	**2494**	**87.7**	**7.8**	**3.1**	**1.1**	**0.3**

* It was not possible to differentiate between the degrees of hearing impairment in children 0–3 years due to the assessment procedures used. Therefore 11.4% reflects the proportion of participants who obtained “refer” as described above (and not necessarily a slight hearing impairment).

**TABLE 2:** Proportion of persons with disabling hearing (%) loss by gender and age categories.

Age Band (Years)	Male (%)	Female (%)	Overall (%)
4–9	1.9	0.0	0.9
10–19	0.0	0.6	1.1
20–29	1.5	1.4	1.9
30–39	9.4	2.1	3.8
40–49	3.7	6.5	10.4
50–59	18.5	10.5	10.6
60+	29.0	11.1	17.9
**Overall**	**5.1**	**3.1**	**4.6**

### Disabling hearing impairment

The prevalence of disabling hearing impairment amongst individuals who participated in this study was 4.57% (95% CI: 3.75% – 5.39%). As a group, males and participants older than 60 years old had a higher proportion of individuals with disabling hearing impairment than other groups of participants.

### Unilateral hearing loss

The prevalence of unilateral hearing impairment in this study was 2.3% (95% CI: 1.71% – 2.89%). Tympanometry assessment also showed that participants in the 0–3 and 4–9 year old age categories had higher rates of abnormal tympanometry findings than other age categories. Refer to Table 3 for laterality of hearing impairment and tympanometry findings.

**TABLE 3:** Laterality of hearing loss & Tympanometry.

Age Band (Years)	Unilateral HI (%)	Bilateral HI (%)	Bilateral Type B* Tympanogram (%)
0–3	2.4	11.5	16.1
4–9	1.4	4.2	5.6
10–19	0.4	2.5	1.8
20–29	2.9	7.9	2.3
30–39	3.1	13.1	0.8
40–49	3.7	21.5	4.4
50–59	1.2	32.9	4.7
60+	6.4	38.9	2.1

**Overall**	**2.3**	**12.3**	**3.4**

* A ‘type B’ tympanogram curve is indicative of a non-compressible fluid within the middle ear space (otitis media), tympanic membrane perforation, or debris within the external ear canal (cerumen).

*Source*: Mikolai, T.K., Duffey, J., & Adlin, D. (2006). A guide to tympanometry for hearing screening: Maico Diagnostic. Retrieved January 23, 2014 from http://www.maico-diagnostic.com/eprise/main/_downloads/com_en/Documentation/Guide.Tymp.pdf

Association between hearing impairment and the following factors was investigated; age, gender, level of education, a family history of hearing impairment, prior episode of stroke (CVA), a self-reported history of hypertension, a self-reported history of diabetes, a prior history of respiratory difficulties, whether participant smokes or not, and a prior history of head and neck trauma. The results of this study showed that increased age, male gender, a family history of hearing impairment, a self-reported history of hypertension, and a prior history of head and neck trauma were associated with hearing impairment (*p* < 0.05) (refer to Table 4 below for more detailed information, including Odds Ration [OR]).

**TABLE 4:** Factors associated with hearing loss.

Hearing loss	Odds ratio (95% CI)	*p*-value
Age	1.03 (1.02, 1.04)	0.001*
Gender	1.62 (1.08–2.41	0.032*
Education	0.82 (0.52, 1.30)	0.404
Family History	3.02 (1.93, 4.73)	0.001*
CVA stroke	0.49 (0.19, 1.24)	0.131
Hypertension	2.05 (1.15, 3.65)	0.015*
Diabetes	0.56 (0.26, 1.20)	0.139
Smoker	1.30 (0.81, 2.08)	0.273
Respiratory difficulties	1.36 (0.76, 2.41)	0.296
Head & Neck Trauma	1.94 (1.00, 3.78)	0.05*

* statistically significant (*p* = 0.05).

## Discussion

The primary aim of this study was to estimate the prevalence of hearing impairment in selected health sub-districts within the Cape Town Metropolitan area. Using the WHO grading of hearing impairment classification (WHO, 2014), it was estimated that overall prevalence of hearing impairment in this study was 12.35% (95% CI: 11.06% – 13.64%). Age, gender, a family history of hearing impairment, a self-reported history of hypertension, and a history of prior head and neck trauma were main factors associated with hearing impairment.

While the estimates of the prevalence of hearing impairment in this study were consistent with that of Stevens *et al.*, (2011), it was noted that it was slightly lower than prevalence rates reported in previous studies which used the WHO Ear and Hearing Disorders Survey protocol (WHO, 1999). For instance, a study conducted in Brazil (Béria *et al.*, 2007) reported prevalence rates of 26.1% (overall hearing impairment) and 6.8% (disabling hearing impairment). An Egyptian study by Abdel-Hamid, Khatib, Aly, Morad, and Kamel (2007) reported prevalence rates of 16.0% (overall hearing impairment) and 2.9% for disabling hearing impairment, whilst a study by Westerberg *et al.* (2008) done in Uganda also reported much higher prevalence rates for disabling hearing impairment than reported in the current study: 11.7% in adults and 10.2% in children respectively.

It is generally difficult to compare prevalence estimates across different studies even when using similar protocols and definitions of hearing impairment. The difficulty in such comparisons stems from differences in demographic characteristics of participant cohorts and study contexts (Lin, Thorpe, Gordon-Salant and Ferrucci (2011b). Therefore, it was expected that prevalence estimates in this study would be somewhat different from those of other studies. However, part of the reasons for differences in the prevalence rates reported in previous studies and the current study may also have to do with the criteria used to ascertain the degree of hearing impairment in children younger than four years old. It was not clear how some of these studies confirmed the degree and type of hearing impairment in children. For instance, the Brazilian study only used pure tone audiometry (and did not use tympanometry) and therefore would have had difficulties in confirming hearing impairment in participants in this age category. The Egyptian study (Abdel-Hamid *et al.*, 2007) on the other hand, deviated from the recommended WHO Ear and Hearing Disorders protocol and used only otoacoustic emission and tympanometry to assess hearing impairment, which makes it extremely difficult to ascertain degree and type of hearing impairment across all age categories.

Consistent with the findings of previous studies (Lin *et al.*, 2011b; McMahon, Kifley, Rochtchina, Newall & Mitchell, 2008; Saunders *et al.*, 2007), this study found that increasing age was associated with hearing impairment. In one American study, Lin *et al.*, (2011b) reported that about two thirds of adults aged 70 years and older had some degree of hearing impairment. Regarding association between gender and hearing impairment, previous research studies (Caban, Lee, Gómez-Marín, Lam & Zheng, 2005; Smeeth *et al.*, 2002) have reported a higher prevalence of hearing impairment in males than females, and the same trend was also observed in this study. It is worth noting though that gender differences in the prevalence of hearing impairment has been reported even in populations with no evidence of noise-induced hearing loss (Pearson *et al.*, 1995) therefore this difference cannot simply be attributed the fact that men generally tend to be exposed to noisy activities.

A family history of hearing impairment and a history of head and neck trauma were other factors that were found to be associated with hearing impairment in this study, which is also consistent with the findings of previous studies (Fitzgerald, 1996; McMahon *et al.*, 2008; Ottaviano *et al.*, 2009). In an Australian study involving 2669 individuals aged 50 years and older, McMahon *et al.*, (2008) reported that a family history of hearing impairment (especially a maternal family history of hearing impairment) was strongly associated with moderate to severe age-related hearing impairment. With respect to a history of head and neck trauma, hearing loss following trauma to the head and neck areas has been documented in literature (Fitzgerald, 1996; Ottaviano *et al.*, 2009).

Results indicate that participants who reported a history of hypertension, in this study, were twice as likely to have hearing impairment as participants who did not report hypertension. This finding was consistent with those of previous studies (Chang *et al.*, 2011; Chao, 2004; Fransen *et al.,* 2008; Kakarlapudi, Sawyer & Staecker 2003). An unexpected finding in this study was the lack of statistically significant association between a history of diabetes and hearing impairment. This is in spite of the fact that there are now several studies that have reported a plausible association between diabetes and hearing impairment (Jáuregui-Renaud, Sánchez, Ibarra Olmos & González-Barcena, 2009; Pemmaiah & Srinivas, 2011; Thimmasettaiah & Shankar, 2012) Possible reasons for the apparent lack of association between diabetes and hearing impairment may have to do with the small number of participants who reported a history of diabetes in this study. Some of the participants may not have been aware that they are diabetic or may have simply chosen not to disclose their diabetes status.

The findings of this study must be interpreted with caution given its methodological limitations. These included unfavourable testing environments as audiological assessment done outside of audiometric booths is bound to be challenging. Specific to this study, the fact that the test environment varied from one household to the next, introduced a further challenge to this study. However, the study protocol was very stringent regarding ambient noise levels that were considered acceptable for a hearing assessment to be conducted. For instance, if the environment was found to be too noisy (i.e. ambient noise >55 dB) then a hearing assessment was not done at all or was terminated. Furthermore, an analysis of the results also revealed that there was no association between a participant being diagnosed with hearing impairment or having a particular degree of hearing impairment and the level of noise in the room.

Another limitation of this study was the small sample size which compromised the representativeness of the study sample. The calculated sample for this study was 5924 participants. However, only 42% (2494 participants) of this sample was achieved during this study. Data collection for this study had to be terminated prematurely before achieving the required sample size due to a flare-up of gang-related violence in the areas selected for this survey. This may have led to the resultant study sample being biased in favour of female participants and participants under the age of 20 years old, and this may have potentially introduced a bias that could have led to an underestimation of the prevalence of hearing impairment in this study. However, despite a possibly biased sample, the trends revealed by the data in this study were consistent with those reported in previous studies, i.e. more hearing impairment in males than females, as well as an association between hearing impairment and advanced age.

## Conclusions

This study showed that the prevalence of hearing impairment in the communities surveyed is much higher than current estimates obtained through national census. Main factors associated with hearing impairment were also identified. Therefore, in spite of its limitations, this study represents one of the few population-based surveys of hearing impairment done in South Africa. The findings of this study could therefore be useful in planning for prevention and interventions services for hearing impairment, especially in the Cape Town Metropolitan area. It is also hoped that the findings of this study will stimulate interest among the South African research community to conduct similar research studies in other parts of the country (especially in rural areas) to obtain a more complete picture of the magnitude of hearing impairment on a national scale. This will yield information that could be used to lobby for resources that are required for prevention and intervention services for hearing impairment.

## Appendix 2

### Instrumentation & Assessment Procedures

#### Instrumentation

- Otoscope Heinne S700 mini
- GSI 39 Tympanometer Screener
- GSI 17 Screening Audiometer
- GSI AUDIOscreener+
- KUDUwave 5000 Audiometer
- Bruel & Kjær 2239 Type II Integrating Sound Level meter (Denmark)
- WHO/PDH Ear and Hearing Disorders Examination Form (Version 7.1A)

#### Assessment procedures

##### Measurement of noise levels

Measurement of ambient noise level in the room was measured using Bruel & Kjær 2239 Type II Integrating Sound Level meter (Bruel & Kjær, Denmark) prior to the start of assessment. If noise levels were deemed suitable for pure tone hearing screening (≤50 dBA ±5dBA) then the researcher proceeded to interview participants to get background information about their hearing/auditory status (i.e. case history information) followed by audiometric assessment.

##### Audiometric assessment

All audiometric equipment used in this study were calibrated prior to the commencement of this study and were used exclusively in this study for the duration of the survey. Furthermore, biological calibration of each piece of equipment was performed every morning before the equipment was used. Audiometric assessment were conducted by trained research assitants (all senior audiology students) under close supervision of a qualified audiologist. Audiometric testing was only done when the ambient noise in the room was deemed suitable for a hearing assessment. The following assessment procedures were followed:

***Individials** ≥ 4 years old*; otoscopic examination was conducted using Heinne S700 mini otoscope (Heinne Optotechnic, Germany) to view and assess the external ear canal and tympanic membrane status. Otoscopic examination was followed by tympanometry screening using Grason-Stadler (GSI 39 screening tympanometer (GSI, Minnesota, United States of America) to check mobility of the tympanic membrane and assess middle ear statusand pure tone audiometry screening. Hearing status was screened using GSI 17 portable audiometer (GSI, Minnesota, United States of America) with standard TDH 39 headphones fittted with Amplivox Audiocups (Amplivox Limited, United Kindom). A screening level of 30 dB HL (with a 60 dB HL 1 kHz as the first tone presented) at the following frequencies; 0.5, 1, 2 & 4 kHz was used and audiometric test started on the ear that the participant considered their best ear. Participants were asked to raise their hands everytime they hear the test tone. Partcipant was considerd to have “passed” the hearing screening (i.e. no hearing impairment) if they responded to at least 3 out of the 4 test frequencies in each ear and a “refer” result (i.e. hearing impairment suspected) was when the participants failed to repond to at least 2 of the test frequencies in at least one ear.

Individuals who obtained “refer” results underwant further assessment (diagnostic) using a KUDUwave 5000 audiometer (GeoAxon, Pretoria, South Africa) to confirm the degree and type of hearing impairment. This audiometer was selected because it is capable of live monitoring of environmental noise and it also has enhanced noise attenuation capability through the use of a combination of insert earphone and circumaural earphone which has implications for accuracy of test results (Swanepoel, Mngemane, Molemong, Mkhwanazi & Tushini, 2010b). Furthermore, it has been shown to yield test results that a comparable to those that are obtained in a standard sound treated environment (Maclennan-Smith, Swanepoel de & Hall, 2013; Swanepoel, Koekermoer & Clark, 2010a).

*For children* ≤ *3 years old;* the following assessment procedures were followed; otoscopic examination (Heinne S700 mini otoscope), tympanometry screening (GSI 39 screening tympanometer [GSI, Minnesota, United States of America]). Distortion Product Otoacoustic Emissions (DPOAE) screening using GSI AUDIOscreener+ (GSI, Minnesota, United States of America) was used instead of pure tone audiometry. A child was considered to have “passed” the hearing screening if a DPOAE response was obtained to at least 2 of the 3 screening frequencies (1, 2 & 3 kHz) plus a Type A tympnogram in both ears. A “refer” results was no response to at least 2 of the screening frequencies with or without an abnormal tympanometry results in at least one ear.

All groups of partcipants (i.e ≤ 3 years old or ≥4 years old), with abnormal audiometric test results following the hearing screening and or diagnostic assessment were referred to the nearest primary health care facility to initiate the process of getting appropriate services for their hearing impairment. All assessment information was recorded on the relevant sections of the WHO/PDH Ear and Hearing Disorders Examination Form (Version 7.1A) (WHO, 1999).

#### Classification of hearing loss

Disabling hearing impairment in this study was defined as a permanent unaided threshold level in the better ear of ≥ 41 dB HL (for adults) and permanent unaided threshold level in the better ear of ≥31 dB or (children younger than 15 years old) (refer to Table 1B below).

**TABLE 1-A2:** WHO Criteria for grading of hearing loss.

Grade of Impairment	Corresponding audiometric ISO value	Performance	Recommendations
0 - No impairment	25 dB or better (better ear)	No or very slight hearing problems	
1 - Slight impairment	26–40 dB (better ear)	Able to hear and repeat words spoken in normal voice at 1 meter	Counselling. Hearing aids may be needed
2 - Moderate impairment	41–60 dB (better ear)	Able to hear and repeat words spoken in raised voice at 1 meter	Hearing aids usually recommended
3 - Severe impairment	61–80 dB (better ear)	Able to hear some words when shouted into better ear	Hearing aids needed, If no hearing aids available, lip reading and signing should be taught
4 - Profound impairment including deafness	81 dB ore greater (better ear)	Unable to hear even at shouted voice	Hearing aids may help understanding words. Additional rehabilitation needed. Lip reading and sometimes signing essential

Grades 2, 3 and 4 are classified as disabling hearing imapirment. The audiometric ISO values are averages of values at 500, 1000, 2000 and 4000 Hz.

*Source*: World Health Organization. (2014). *Grades of hearing impairment*. Retrieved October 10, 2014, from http://www.who.int/pbd/deafness/hearing_impairment_grades/en/index.html
